# A Forehead Wearable Sensor for the Objective Measurement of Chronic Pain

**DOI:** 10.3390/ijerph192417041

**Published:** 2022-12-19

**Authors:** Marcus Orzabal, Ramo Naidu, Kasra Amirdelfan, Alireza Akhbardeh

**Affiliations:** 1 CereVu Medical Inc., 688 Missouri Street, San Francisco, CA 94107, USA; 2 California Orthopedics & Spine, 2 Bon Air Road, Larkspur, CA 94939, USA; 3 IPM Medical Group, 450 N Wiget Lane, Walnut Creek, CA 94598, USA

**Keywords:** chronic pain, cerebral optical spectrometry, objective pain assessment, algorithms

## Abstract

Chronic pain impacts one in five Americans and is difficult to manage, costing ~USD 600 billion annually. The subjective experience of pain is a complex processing of central nervous system input. Recent advances in magnetic resonance imaging revealed the prefrontal cortex as vital to the perception of pain and that changes in the cerebral hemodynamics can be used to detect painful sensations. Current pain monitoring is dependent on the subjective rating provided by patients and is limited to a single time point. We have developed a biomarker for the objective, real-time and continuous chronic pain assessment using proprietary algorithms termed ROPA and cerebral optical spectrometry. Using a forehead sensor, the cerebral optical spectrometry data were collected in two clinical sites from 41 patients (19 and 22, respectively, from sites 1 and 2), who elected to receive an epidural steroid injection for the treatment of chronic pain. Patients rated their pain on a numeric rating scale, ranging from 0–10, which were used to validate the ROPA objective pain scoring. Multiple time points, including pre- and post-procedure were recorded. The steroid injection was performed per standard medical practice. There was a significant correlation between the patient’s reported numeric rating scale and ROPA, for both clinical sites (Overall ~0.81). Holding the subjective pain ratings on a numeric rating scale as ground truth, we determined that the area under the receiver operator curves for both sites revealed at least good (AUC: 64%) to excellent (AUC > 98%) distinctions between clinically meaningful pain severity differentiations (no/mild/moderate/severe). The objective measure of chronic pain (ROPA) determined using cerebral optical spectrometry significantly correlated with the subjective pain scores reported by the subjects. This technology may provide a useful method of detection for the objective and continuous monitoring and treatment of patients with chronic pain, particularly in clinical circumstances where direct assessment is not available, or to complement the patient-reported pain scores.

## 1. Introduction

Chronic primary pain, or pain lasting longer than 3 months, is a debilitating condition affecting 100 million patients in the United States with a total incremental economic burden of USD 600 billion per year [1,2]. Despite decades of research, our understanding of chronic pain remains incomplete. Current methods of chronic pain determination are limited to subjective pain rating scales, typically ranging from 0 (no pain) to 10 (worst pain imaginable) [3]. The subjective, self-reporting assessments, such as the numerical rating scale (NRS), verbal rating scale, and visual analog scale (VAS), remain the current gold standard for patients who are conscious, have precise understanding, and verbal expression [4]. However, these methods reduce the power of pain monitoring due to the subjective nature of the rating system, psychological/environmental factors, the ability to collect only a single time point, and the lack of physiological parameters that correspond to pain perception [5,6]. Thus, it is imperative that the future of pain monitoring implement an objective pain measurement system that offers real-time and continuous evaluations to personalize therapeutics, reduce the over-prescription of pain medications, and improve the remote patient monitoring.

Pain is a subjective experience with both physical and psychological components [7]. The fundamental understanding of nociceptive processing encompasses a network of anatomical pathways originating in the periphery and ascending through the spinothalamic tracts. There is also an increased knowledge about how various regions of the brain process the nociceptive and painful sensory information [8]. This includes the interconnectivity of the different brain regions that, in combination, generate and provide the complete physical and emotional experience of pain itself. Functional imaging studies have demonstrated several key brain regions involved in the processing of pain, including the prefrontal regions, anterior cingulate cortex, somatosensory cortex, amygdala, and others [9,10]. This work has elucidated the relationships between the activity and connectivity between the different brain regions and the painful experience that individuals subjectively describe, such as the pain quality and intensity. Although cumbersome, a functional MRI (fMRI) of the prefrontal cortex in subjects with chronic low back pain show strong correlations between the prefrontal cortex activity and intensity/duration of pain [11,12].

Based on the findings of this prior cerebral nociceptive processing research, we herein propose a more feasible method to capture real-time objective pain assessments (ROPAs) utilizing cerebral optical spectrometry to measure the hemodynamic changes in a population suffering from chronic pain. The ROPA measurement is computed using specialized cerebral sensors, signal processing, and predictive model algorithms. The primary objective of the study is to determine the changes in the cerebral nociceptive hemodynamic response (blood oxygenation/flow) before and after an interventional pain treatment—an epidural steroid injection. The physiological measurements of the cerebral nociceptive hemodynamic responses will be obtained with a FDA-cleared forehead sensor (Covidien^®^) and will be analyzed offline and compared against subjective measures of pain reported by subjects. Proprietary analysis algorithms were incorporated and developed to understand how the objective measurements correlate with the subject reported pain levels.

## 2. Materials and Methods

### 2.1. Subjects

All methods and procedures were approved by the Western Institutional Review Board, Inc. (Puyallup, WA, USA) under study number 1255984, and IRB tracking number: 20190594. Written informed consent was obtained from all participants before the start of the study. A prospective, observational, single-arm study design was used in which each subject acted as their own control. A total of 51 subjects were evaluated, however 41 subjects were utilized for the analysis in this study. Clinical procedures and data collection were performed at California Orthopedics & Spine (*n* = 19) and IPM Medical Group (*n* = 22). The subjects were recruited for this study, based on the following qualifications: between the age of 18 and 75; diagnosed with primary chronic low back and/or leg pain for at least 6 months; and is a candidate for a transforaminal, epidural steroid injection (ESI) to treat chronic back and/or lower extremity pain, as a standard of care. Participants were excluded from the study if they are allergic to any adhesives/materials used in conjunction with the Covidien^®^ sensor; have a known hypersensitivity to steroids; have a local or systemic infection, local malignancies, bleeding diathesis, congestive heart failure, or uncontrolled diabetes mellitus.

### 2.2. Epidural Steroid Injection

The subjects were placed in a prone position and prepped for the transforaminal ESI per routine sterile precautions. A finger pulse oximeter was applied to the subject to collect the heart rate and SpO_2_ data. An intravenous (IV) catheter was placed, and the subject was sedated with midazolam, as needed, following the standard anesthesia guidelines and at the discretion of the treating physician. Subjects did not receive IV or oral opioids as part of the pre-operative sedation procedure.

The lumbar transforaminal ESI procedure was then performed, per standard medical practice. At clinical site 1, California Orthopedics & Spine, a 6 mg dose of betamethasone was coupled with a local anesthetic of 1 mL of 0.25% bupivacaine for a total of 2 mL at each level. At clinical site 2, IPM Medical Group, a 40 mg dose of triamcinolone was coupled with a local anesthetic of 0.5 mL of 4% Xylocaine at each level—for a maximum total volume of 1 mL administered at each level. The following data variables were collected at the baseline before the procedure, and in recovery after the procedure: the cerebral hemoglobin oxygenation and de-oxygenation; subjective pain assessment via a numeric rating scale (NRS) 0–10; heart rate; and SpO2.

### 2.3. Subjective Pain Ratings

Subjects were instructed to rate their pain on a numeric rating scale ranging from 0–10, at multiple specific time points throughout the visit. The physiological data was captured by an optical spectrometry sensor with a sampling rate of 500 Hz and down sampled at a rate of 1 Hz for further data analysis.

During the course of the study, the subjects received a standard 6 mg dose of betamethasone or 40 mg of triamcinolone were used for the steroid injection at California Orthopedics & Spine and IPM Medical Group, respectively. The subjective verbal numeric rating scale (NRS) ratings were obtained at multiple time points throughout the study with 0 being “no pain” and 10 being “the worst pain imaginable” from initiation of the baseline measurements until the end of the post-procedure recovery period.

The following planned intervention time points were specifically targeted for the data collection during the procedure. Data were collected prior to and after each of these events. Additional information was collected if any other interventions occurred that might elicit a pain response:

1. Procedural baseline data reading once subject is positioned (prior to any intervention);
2. IV catheter insertion;
3. Administration of IV sedation;
4. Local anesthesia needle insertion;
5. Steroid and local anesthetic transforaminal injection;
6. End of procedure data reading (following completion of all interventions).

At the conclusion of the ESI procedure, the level of sedation the subject received during the procedure was documented. Subjects continued to wear the Covidien^®^ sensor after being moved to a recovery area, and the cerebral optical spectrometry readings were taken during the post-procedural recovery period. The subjective NRS pain scores were reported verbally and documented on appropriate case report forms (CRFs). Any post-procedure analgesics administered were documented. Any adverse events related to the device that occurred during the procedure or prior to discharge were documented on the adverse events CRF.

### 2.4. Optical Spectrometry

The skin on the forehead was prepped with an alcohol swab, and following the directions on the packaging, a commercially available Covidien^®^ sensor (Medtronic, Nellcor™, Pleasanton, CA, USA) was applied and secured to the forehead with a headband. The sensor was then connected to the investigational device.

**Figure 1:**
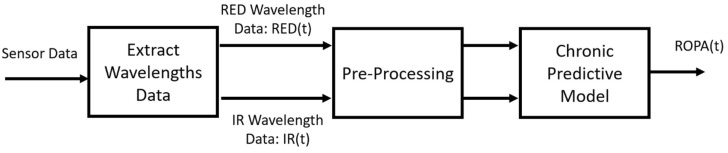
The pipeline for the signal processing applied to calculate the ROPA index.

Using signal processing and predictive model algorithms, a “real-time objective pain assessment” (ROPA, CereVu Inc., San Francisco, CA, USA) was computed. The data were processed following the steps detailed in our previous investigation and can be seen outlined in Figure 1. Briefly, the calibrations are performed to reduce the ambient light and account for the subject’s skin tone. The cerebral optical spectrometry uses RED and infra-red light to target the cerebral vasculature [13,14]. The RED and infra-red light signals are extracted and pre-processed before our novel predictive model algorithm is applied. The final step is to align the time series of the experiment to compare the subjective NRS ratings and the ROPA score correlation. All ROPA scores were calculated offline by an investigator who was blinded from any recorded study events.

### 2.5. Data Analysis

All data analyses were carried out using MATLAB for Mac. Since minor motion artifacts were expected in the subject population, we examined the ROPA readings time course in windows of 30 s prior to 30 s after the subject-reported pain score, for the data quality check. The data points were not included in the analysis if gaps of more than 10 s were present within the 1-min window, indicating events, such as the sensor dislocation. Exclusion of these data points also decreased the potential lag time/hysteresis between the subjects’ reported pain and the ROPA scores because there was not a continuous subjective pain recording.

The correlations were calculated between the subjective pain ratings (NRS) and the objectively measured chronic pain scoring (ROPA) for both clinical sites (separate and pooled). This was calculated using Pearson’s correlation coefficients.

The receiver operating curves (ROC) were used to evaluate the performance of the ROPA scoring algorithms. The area under the ROC (AUC) represents the ability to differentiate between the clinically meaningful cut-off values (no/mild–moderate–severe pain) in the subjects. The comparisons we chose to make were between:

1. No/mild pain (NRS ≤ 3) vs. moderate/severe pain (NRS > 3);
2. No/mild/moderate pain (NRS ≤ 6) vs. severe pain (NRS > 6);
3. No/mild pain (NRS ≤ 3) vs. severe pain (NRS > 6).

The AUCs were classified as having either a good accuracy (70–79%), a very good accuracy (80–89%) or an excellent (greater than 90%).

## 3. Results

### 3.1. Subjects

All patients who elected to receive the ESI procedure completed the study. The demographics of the subjects are displayed in Table 1. From clinical site 1 (California Orthopedics & Spine), 26 subjects were enrolled, however, only data from 19 subjects were usable and data from seven subjects were lost due to severe motion artifacts, sensor displacement issues, or human error. From clinical site 2 (IPM Medical Group), 25 subjects were enrolled, however, only data from 22 subjects were usable and data from three subjects were lost due to severe motion artifacts or human error.

### 3.2. Correlation Analysis

**Figure 2:**
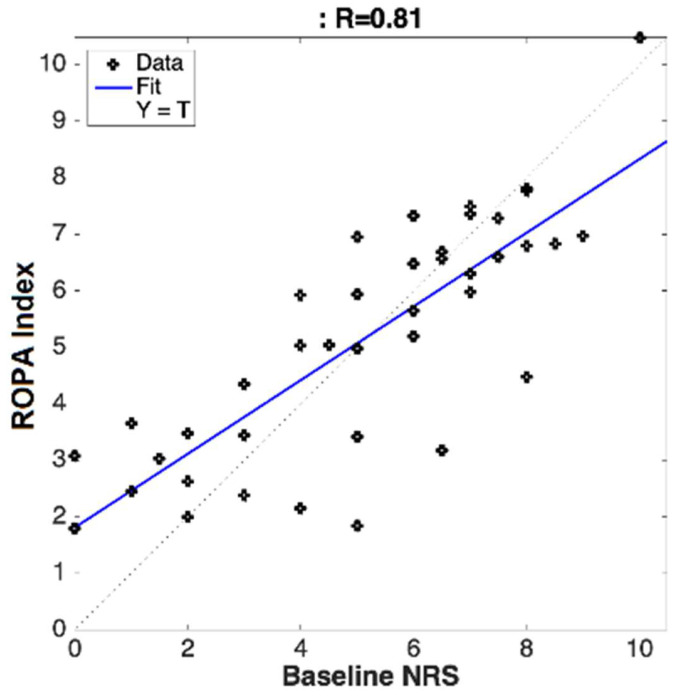
Linear regression between the subject-provided numeric pain rating scale (NRS) at baseline (before the epidural steroid injection) and the objectively measured changes in the physiological parameters responding to pain (ROPA Index).

**Figure 3:**
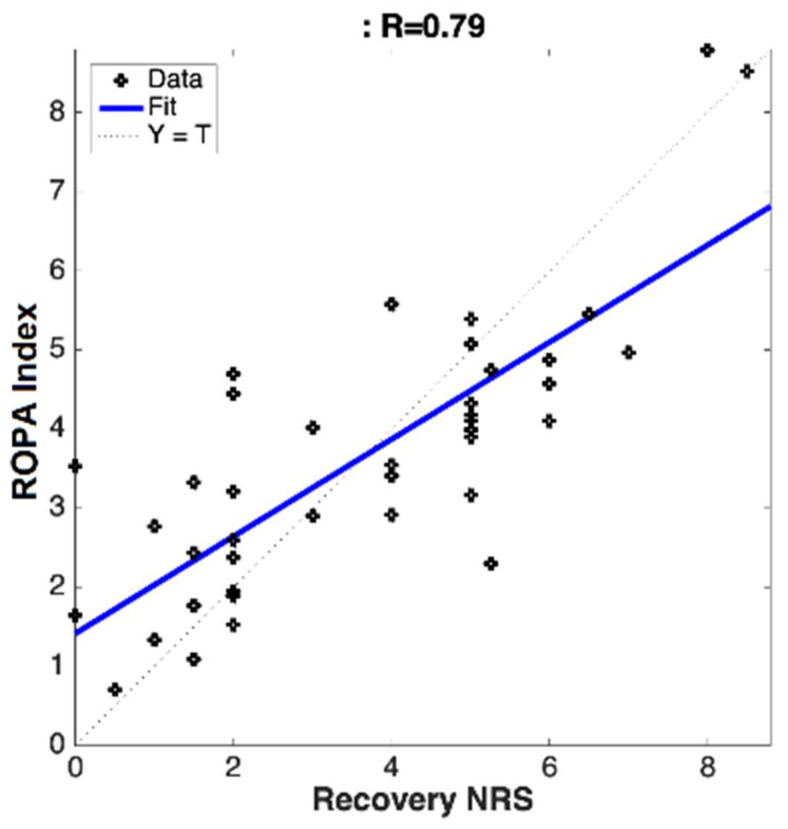
Linear regression between the subject-provided numeric pain rating scale (NRS) in the recovery room (after the epidural steroid injection) and the objectively measured changes in the physiological parameters responding to pain (ROPA Index).

A linear correlation analysis of the pooled data from both clinical sites (Figure 2 and Figure 3) gave a linear correlation strength (R) value of R = 0.81 for the subjects at the baseline, and R = 0.79 for the subjects during the recovery period.

### 3.3. ROC Analysis

**Figure 4:**
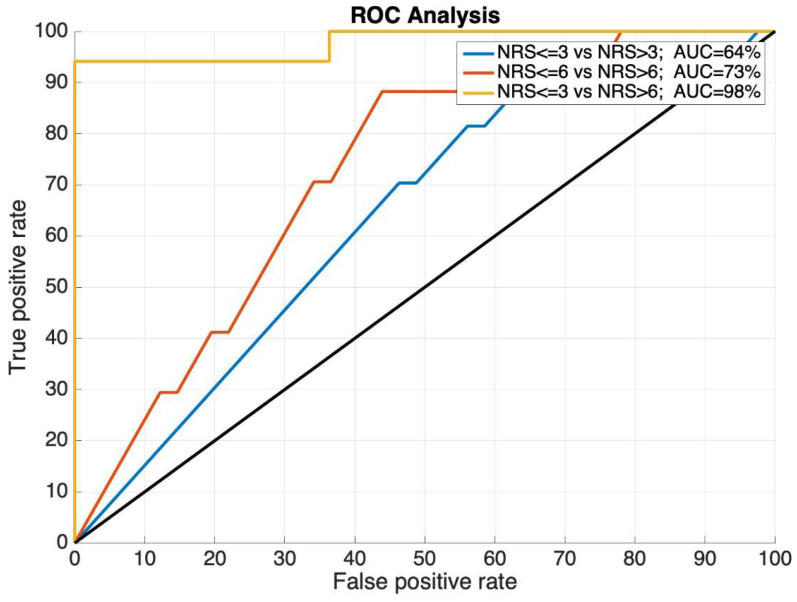
Baseline period receiver operating curves for the numeric rating scale (NRS) ≤ 3 and NRS > 3 (blue line, AUC = 64%); NRS ≤ 6 and NRS > 6 (orange line, AUC = 73%); and NRS ≤ 3 and NRS > 6 (yellow line, AUC = 98%). AUC values, used as a performance indicator, were excellent (AUC > 90%).

**Figure 5:**
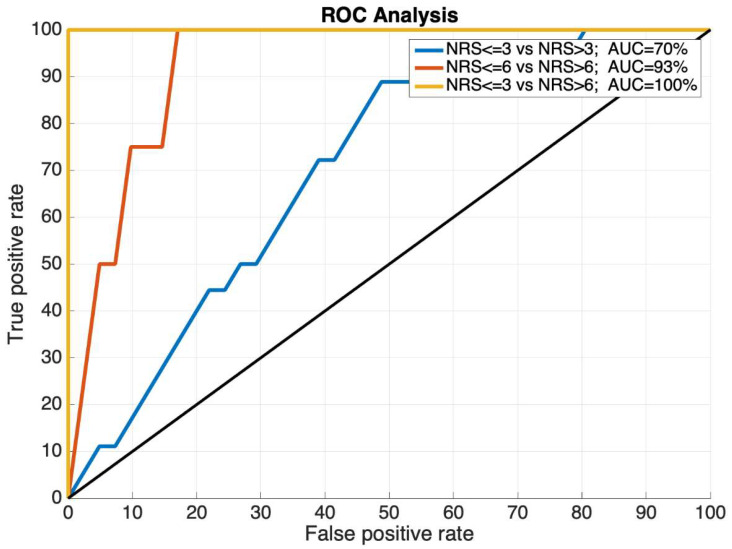
Recovery period receiver operating curves for the numeric rating scale (NRS) ≤ 3 and NRS > 3 (blue line, AUC = 70%); NRS ≤ 6 and NRS > 6 (orange line, AUC = 93%); and NRS ≤ 3 and NRS > 6 (yellow line, AUC = 100%). AUC values, used as a performance indicator, were excellent (AUC > 90%).

The ROC analysis displayed accuracies that ranged from just below good (64%) to excellent (100%), as indicated by the AUC values. Figure 4 displays the ROCs for the subjects at the baseline and Figure 5 displays the ROCs for the subjects during the recovery period. The baseline AUCs ranged from less than good (no/mild pain vs. moderate/severe pain) to very good (no/mild/moderate pain vs. severe pain), or excellent (no/mild pain vs. severe pain). The recovery AUCs ranged from good (no/mild pain vs. moderate/severe pain), very good (no/mild/moderate pain vs. severe pain), and excellent (no/mild pain vs. severe pain).

Table 2 summarizes the maximum sensitivity, maximum specificity, and the area under the curve for the investigated ROCs.

## 4. Discussion

Utilizing a novel algorithm, in combination with cerebral optical spectrometry, this study aimed to calculate the real-time, objective, and continuous chronic pain scores for patients suffering from chronic lower back or leg pain, who elected to receive a transforaminal epidural steroid injection. The algorithm computed an individualized ROPA index score, based on the physiological parameters collected from the forehead sensor. It was determined that the calculated ROPA index was significantly correlated to the subjective pain ratings provided by the patients before and after treatment. The data collected in this study have revealed several key findings, including: (1) chronic pain can be objectively measured in real-time and continuously, using noninvasive cerebral optical spectrometry and our proprietary algorithms; (2) cerebral optical spectrometry-based chronic objective pain assessment (ROPA) significantly correlated with the subjectively reported pain; (3) the proprietary algorithm can distinguish between mild, moderate, and severe pain with a high degree of accuracy.

The perception of pain is an extremely complex phenomenon that is attributed to physical and psychosocial factors that produce a unique experience [15]. Individual pain tolerance also contributes to differences in pain perception [16]. Clinical pain assessments are primarily used to qualify the severity, frequency or chronicity of pain, and to inform treatments [17]. The most commonly used method to measure pain in the clinical setting is the NRS [3,4]. However, this method is limited to a single data point; is susceptible to bias due to the subjective aspect of the measurement; and is only viable if the patient is conscious and able to communicate their experience. While subjective pain scales are useful, these limitations highlight the need for a supplemental measurement that is continuous, incorporates the objective physiological parameters, and is easy to interpret.

There are several alternative single-variable methods to subjective pain assessments that are currently being explored, including the heart rate variability and skin conductance. A commercially available heart rate variability-based system has been used to predict NRS ratings in patients upon arrival to a postoperative recovery room, with a good degree of accuracy (89%) [18]. However the heart rate variability-based analgesia nociceptive index in healthy volunteers exposed to expected and unexpected electrical stimulation, did not correlate with the subject pain ratings and could not differentiate between painful and non-painful stimuli [19]. A skin conductance-based pain assessment utilizing the frequency of fluctuations in postoperative patients was able to distinguish between the NRS pain rating of ≤3 and >3 with 88.5% sensitivity and 67.7% specificity [20]. In healthy volunteers exposed to thermal stimuli, skin conductance was a more sensitive indicator of changes in the pain perception within the individual, when compared to the heart rate variation, but was not significantly correlated to the subjective ratings within the group [21]. Conflicting outcomes of heart rate variability-based and skin conductance-based pain assessments therefore raise concern for reliability and necessitate the further investigation of single-variable pain assessment methods.

Multi-variable approaches to pain monitoring may offer a more reliable method to detect and differentiate the pain severity. A study designed to classify noxious vs. non-noxious events during surgery utilized heart rate, heart rate variability, plethysmograph wave amplitude, skin conductance level, and the number of skin conductance fluctuations to develop an objective index of the nociceptive level in patients under general anesthesia [22]. The multi-parameter system was able to distinguish between innocuous and noxious stimuli to a greater degree, compared to the use of any single-parameter analysis. This study demonstrates the value in using a multidimensional index of pain but is limited to variables that may be influenced by an autonomic response to stimuli other than pain. Based on our current understanding of pain perception, a more accurate measure of pain may need to incorporate multiple physiological parameters, as well as the impact of the central nervous system processing.

Breakthroughs in fMRI have elucidated the regions of the central nervous system associated with pain perception [23]. The prefrontal cortex has been identified as a major component in the individual experience of pain and its intensity [24]. Activity in this region of the brain has been shown to increase in the presence of noxious stimuli [25]. The hemodynamic response to this activity can subsequently be evaluated using cerebral optical spectrometry, an inexpensive and non-invasive method of measuring tissue oxygenation that significantly correlates with fMRI [26]. Researchers investigating the efficacy of optical spectrometry in pain perception were able to distinguish the frontal lobe activation and deactivation in the presence of noxious electrical stimuli [27]. This study demonstrates the ability to provide objective information about the subjective experience of pain. We have developed novel algorithms that further examine the cerebral hemodynamic changes to calculate an objective measure of pain intensity.

We herein aimed to demonstrate the utility of our proprietary predictive modeling algorithms by processing extensive cerebral optical spectrometry data, to calculate an objective pain assessment in patients with chronic pain. We have previously demonstrated that cerebral optical spectrometry, combined with our algorithms can be used to identify and classify acute pain with a good degree of accuracy in laboring women [28]. In the current study we have modified our proprietary algorithm to account for chronic pain-induced hemodynamic changes in the prefrontal cortex. We achieved a significant correlation between the calculated ROPA score and the subject-reported pain across multiple sites. The ROPA system was able to distinguish between mild/moderate/severe pain with varying degrees of accuracy and requires further validation. The ROPA index calculated in this study is unique in its ability to objectively quantify chronic pain and may be used as an adjunct measure in the clinical diagnosis, monitoring, and treatment of chronic pain.

### Limitations

Continued research is needed to expand the subject pool used in this study. The novelty of the objective chronic pain assessment algorithm requires further validation in populations experiencing chronic pain of differing intensities, chronicity, and bodily location. Additionally, this study was designed to monitor sedated subjects in a prone position and will require evaluations of fully conscious subjects in a variety of positions. This study utilized the Covidien Max-Fast sensor. For future studies, we will use our newly designed CereVu system which includes a CereVu wireless forehead sensor with an incorporated accelerometer, to account for patient movement, and a fully integrated iOS app.

## 5. Conclusions

Utilizing cerebral optical spectrometry, in combination with our novel algorithm, we were able to provide an objective, real-time, and continuous measure of pain in patients suffering from chronic low back pain, that significantly correlates to the subjective pain ratings with an accuracy ranging from less than good to excellent. Although further validation is needed, this study builds on the previous understanding of pain perception within the central nervous system and offers an alternative approach to chronic pain monitoring.

## 6. Patents

AA is the founder and shareholder of CereVu Medical Inc. CereVu has filed several patent applications to protect our technology. The patent applications include:

U.S. Patent Application (with generic description of algorithms) filed 2 October 2014 with priory date of 11 March 2013, under application number US 14/203,987. This patent application is mainly focused on describing the end-to-end systems and the methods description (sensor, headband, hardware, algorithm, use cases and applications).

PCT-International Patent Application (with generic description of algorithms) filed 3 November 2014 with priory date of 11 March 2013, under application number WO2014164717. This patent application is mainly focused on describing the end-to-end systems and the methods description (sensor, headband, hardware, algorithm, use cases and applications).

Due to the proprietary nature of the algorithms, the methods used to calculate the objective pain scores is not fully disclosed. The present study was conducted to validate the application of these algorithms.

## Figures and Tables

**Table 1:** Subject Demographics.

	Average	Range
Age	60	37–79
Weight (lb)	168	103–245
Sex (m:f)	12:28	
Race	N	
Native American	1	
Caucasian	25	
Latin American	2	
Native Hawaiian/Pacific Islander	0	
Asian	4	
Black	0	
Other	1	
Not Reported	1	
Ethnicity	N	
Hispanic	2	
Non-Hispanic	33	
Not Reported	1	

**Table 2:** Sensitivity, specificity, and the area under the receiver operating curve (AUC) at the baseline and recovery.

Baseline	Sensitivity (%)	Specificity (%)	AUC (%)
VAS ≤ 3 vs. VAS > 3	70.38	53.67	64
VAS ≤ 6 vs. VAS > 6	70.6	66.00	73
VAS ≤ 3 vs. VAS > 6	90	100	98
Recovery			
VAS ≤ 3 vs. VAS > 3	72.20	61.0	70
VAS ≤ 6 vs. VAS > 6	100	83	93
VAS ≤ 3 vs. VAS > 6	100	0	100

## Data Availability

The data that support the findings of this study are available upon request from the corresponding author. The data are not publicly available due to restrictions (e.g., their containing information that could compromise the privacy of the research participants).
